# 2-Amino-6-[(2,6-dichloro­phen­yl)imino]-3-oxocyclo­hexa-1,4-dienecarbaldehyde

**DOI:** 10.1107/S1600536811042619

**Published:** 2011-10-22

**Authors:** Cláudia M. B. Neves, José A. Fernandes, Mário M. Q. Simões, M. Graça P. M. S. Neves, José A. S. Cavaleiro, Filipe A. Almeida Paz

**Affiliations:** a Department of Chemistry, University of Aveiro, QOPNA, 3810-193 Aveiro, Portugal; b Department of Chemistry, University of Aveiro, CICECO, 3810-193 Aveiro, Portugal

## Abstract

The title compound, C_13_H_8_Cl_2_N_2_O_2_, was obtained by the oxidation of diclofenac {systematic name: 2-[2-(2,6-dichloro­phenyl­amino)­phen­yl]acetic acid}, an anti-inflammatory drug, with hydrogen peroxide catalysed by chlorido[5,10,15,20-tetra­kis­(2,6-dichloro­phen­yl)porphyrinato]manganese(III), using ammonium acetate as co-catalyst. The asymmetric unit contains two crystallographically independent mol­ecules of the title compound (*Z*′ = 2). The close packing of individual mol­ecules is mediated by a series of strong and rather directional N—H⋯Cl and N—H⋯O hydrogen bonds, plus weak π–π [distance between the individual double bonds of symmetry-related imino­quinone rings = 3.7604 (13) Å] and Cl⋯O inter­actions [3.0287 (18) Å].

## Related literature

For background to diclofenac oxidation reactions using metalloporphyrins as catalysts, see: Othman *et al.* (2000 ▶). For oxidation of other drugs and other organic compounds by hydrogen peroxide catalysed by metalloporphyrins, see: Othman *et al.* (2000 ▶); Bernadou & Meunier (2004 ▶); Mansuy (2007 ▶); Neves *et al.* (2011 ▶); Simões *et al.* (2009 ▶); Rebelo *et al.* (2004*a* ▶,*b* ▶, 2005 ▶). For crystallographic studies from our research group of compounds with biological activity, see: Fernandes *et al.* (2010 ▶, 2011 ▶); Loughzail *et al.* (2011 ▶). For a description of the graph-set notation, see: Grell *et al.* (1999 ▶).
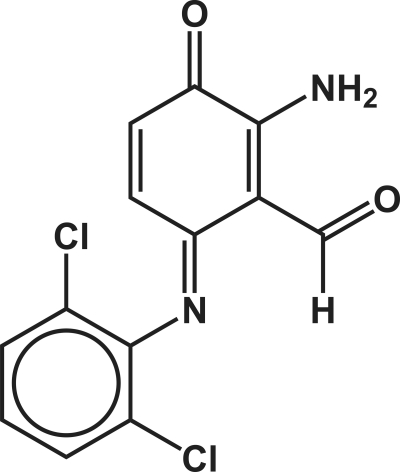


## Experimental

### 

#### Crystal data

C_13_H_8_Cl_2_N_2_O_2_
*M* *_r_* = 295.11
Monoclinic, 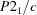

*a* = 17.1738 (14) Å
*b* = 10.5718 (8) Å
*c* = 14.1457 (11) Å
β = 101.192 (5)°
*V* = 2519.4 (3) Å^3^
*Z* = 8
Mo *K*α radiation
μ = 0.51 mm^−1^
*T* = 150 K
0.07 × 0.04 × 0.01 mm

#### Data collection

Bruker X8 KappaCCD APEXII diffractometer
Absorption correction: multi-scan (*SADABS*; Sheldrick, 1997 ▶) *T*_min_ = 0.965, *T*_max_ = 0.995
24324 measured reflections
4600 independent reflections
3466 reflections with *I* > 2σ(*I*)
*R*_int_ = 0.040

#### Refinement

*R*[*F* ^2^ > 2σ(*F* ^2^)] = 0.032
*wR*(*F* ^2^) = 0.085
*S* = 1.02
4600 reflections
355 parameters
6 restraints
H atoms treated by a mixture of independent and constrained refinement
Δρ_max_ = 0.24 e Å^−3^
Δρ_min_ = −0.24 e Å^−3^

### 

Data collection: *APEX2* (Bruker, 2006 ▶); cell refinement: *SAINT-Plus* (Bruker, 2005 ▶); data reduction: *SAINT-Plus*; program(s) used to solve structure: *SHELXTL* (Sheldrick, 2008 ▶); program(s) used to refine structure: *SHELXTL*; molecular graphics: *DIAMOND* (Brandenburg, 2009 ▶); software used to prepare material for publication: *SHELXTL*.

## Supplementary Material

Crystal structure: contains datablock(s) global, I. DOI: 10.1107/S1600536811042619/tk2799sup1.cif

Structure factors: contains datablock(s) I. DOI: 10.1107/S1600536811042619/tk2799Isup2.hkl

Supplementary material file. DOI: 10.1107/S1600536811042619/tk2799Isup3.cml

Additional supplementary materials:  crystallographic information; 3D view; checkCIF report

## Figures and Tables

**Table 1:** Hydrogen-bond geometry (Å, °)

*D*—H⋯*A*	*D*—H	H⋯*A*	*D*⋯*A*	*D*—H⋯*A*
N2—H2*X*⋯Cl1^i^	0.92 (1)	2.60 (1)	3.4590 (18)	156 (2)
N2—H2*Y*⋯O1	0.92 (1)	2.08 (2)	2.722 (2)	126 (2)
N2—H2*Y*⋯O4^i^	0.92 (1)	2.26 (2)	2.933 (2)	130 (2)
N4—H4*X*⋯O1^i^	0.93 (1)	2.04 (1)	2.916 (2)	155 (2)
N4—H4*Y*⋯O3	0.92 (1)	2.01 (2)	2.666 (3)	127 (2)

Symmetry code: (i) 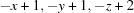.

**Fig. 1.**
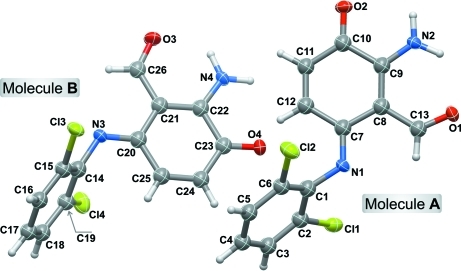
Asymmetric unit of the title compound showing the two crystallographic independent molecular units coined A and B. Displacement ellipsoids are drawn at the 50% probability level and the atomic labeling is provided for all non-hydrogen atoms. Hydrogen atoms are represented as small spheres with arbitrary radius.

**Fig. 2.**
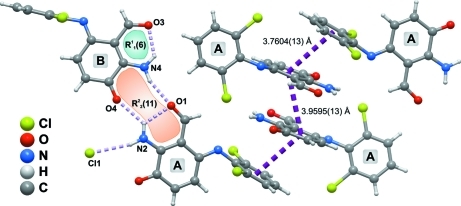
Supramolecular contacts interconnecting adjacent molecules A and B of the title compound. On the left the strong N—H···O and N—H···Cl hydrogen bonds can be grouped into two graph set motifs: R11(6) and R22(11). On the right, weak π–π contacts involving double bonds of the iminoquinone and aromatic rings further ensure supramolecular connections among A molecules. For geometric details on the represented hydrogen bonds see Table 1. Symmetry transformations used to generate equivalent atoms have been omitted for simplicity.

**Fig. 3.**
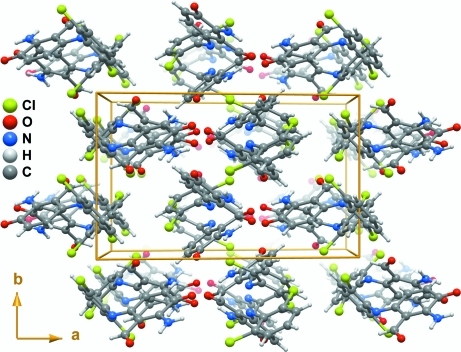
Crystal packing of the title compound viewed in perspective along the [001] direction of the unit cell. Supramolecular interactions have been omitted for clarity.

## supplementary crystallographic information

### Comment

The possibility of using synthetic metalloporphyrins as biomimetic catalysts, which are able to mimic cytochrome P450 enzymes, has attracted the interest of many research groups (Othman *et al.*, 2000; Bernadou *et al.*, 2004; Mansuy, 2007), including ours (Neves *et al.*, 2011; Simões *et al.*, 2009; Rebelo *et al.*, 2004*a*, 2004*b*, 2005). In particular, our current research is focused on the preparation of putative metabolites by the *in vitro* oxidation of drugs. These studies will allow the production of metabolites in the amounts of milligrams, the isolation and identification of unstable intermediates and the understanding of the mechanism of action of drugs (Bernadou *et al.*, 2004). The title compound, C_13_H_8_Cl_2_N_2_O_2_, was obtained by the oxidation of 2-(2-(2,6-dichlorophenylamino)phenyl)acetic acid (diclofenac), an anti-inflammatory drug, with hydrogen peroxide catalysed by chloro[5,10,15,20-tetrakis(2,6-dichlorophenyl)porphyrinato]manganese(III) using ammonium acetate as co-catalyst. Following our on-going interest on the structural features of compounds with biological activity (Fernandes *et al.*, 2010, 2011; Loughzail *et al.* 2011) here we wish to report the crystal structure of the oxidation product of diclofenac.

The asymmetric unit of the title compound comprises two whole molecules of C_13_H_8_Cl_2_N_2_O_2_ (Fig. 1). A comparison between the geometrical features of the two molecules reveals that bond distances and angles involving equivalent atoms are very similar (deviations smaller than 0.012 Å and 1.6°, respectively). There are, however, some considerable differences concerning torsion angles, namely those subtended by the two six-membered rings in each molecule: 71.97 (10)° for molecule A and 75.89 (10)° for molecule B.

The crystal is rich in supramolecular interactions, namely π–π (involving the individual double bonds of the iminoquinone rings), Cl···O and hydrogen bonding interactions. The π–π interactions occur between pairs of molecules A involving the aromatic and the iminoquinone rings, or two iminoquinone rings [distance between centroids of 3.7604 (13) and 3.9595 (13) Å, respectively - purple dashed bonds in Figure 2]. A pair of B molecules also exhibits a short Cl···O interaction (Cl···O distance 3.0287 (18) Å, not shown].

The two crystallographically independent molecules have a different behaviour concerning the hydrogen bonding network in which they are involved (Figure 2 and Table 1 for geometric details). Molecule A is engaged in a bifurcated N—H···(O,O) hydrogen bond, which is shared by the aldehyde group (intramolecular) and the ketone group of a neighbouring B molecule. The remaining N—H moiety of molecule A donates the hydrogen atom to a Cl atom of a neighbouring A molecule. The NH_2_ group of molecule B participates in two N—H···O_aldehyde_ interactions, of which one is intramolecular and the other occurs with molecule A. The hydrogen bonds form discrete clusters (violet dashed lines in Fig. 2) which can be described as the merging of two rings with a graph set notations *R*^1^_1_(6) and *R*^2^_2_(11), respectively (Grell *et al.*, 1999).

Unequivocally, the strongest connection among adjacent molecules corresponds to that of the latter graph set, which leads to the formation of dimers as depicted in Fig. 2. The crystal packing is, thus, promoted by the close packing of such dimers: firstly, and mediated by the aforementioned weak π–π contacts, dimers form columnar arrangements along the *c*-axis of the unit cell. Secondly, columns pack in the *ab* plane in a typical brick-wall-type fashion as depicted in Fig. 3.

### Experimental

All chemicals were purchased from commercial sources and were used as received without further purification.

The oxidation reactions were carried out using 0.1 mmol of diclofenac (sodium salt, Sigma-Aldrich), 1.33 µmol of chloro[5,10,15,20-tetrakis(2,6-dichlorophenyl)porphyrinato]manganese(III) ([Mn(TDCPP)Cl, as catalyst) and 15 mg of co-catalyst (ammonium acetate, Fluka) in CH_3_CN: H_2_O (10:1), in a total volume of 2.0 ml under normal atmosphere at 30 °C. The oxidant employed was aqueous hydrogen peroxide 30% (*w*/*w*) (Riedel-de Haën) diluted 1:5 in CH_3_CN. The oxidant (0.05 mmol) was added to the reaction mixture every 15 min. After 8 h of reaction, the mixture was extracted with dichloromethane and purified by preparative TLC using the same solvent as eluent. The product was dissolved in a minimum amount of dichloromethane and crystallized in hexane at around -16 °C to isolate crystals of the title compound.

### Refinement

Hydrogen atoms bound to carbon were placed at their idealized positions and were included in the final structural model in riding-motion approximation with C—H = 0.95 Å. The isotropic thermal displacement parameters for these hydrogen atoms were fixed at 1.2×*U*_eq_ of the respective parent carbon atom.

Hydrogen atoms bound to nitrogen were directly located from difference Fourier maps and included in the final structural model with the N—H and H···H distances restrained to 0.95 (1) and 1.55 (1) Å, respectively. The *U*_iso_ of these hydrogen atoms was fixed at 1.5×*U*_eq_ of the nitrogen atom to which they are attached.

### Crystal data

**Table:**

C_13_H_8_Cl_2_N_2_O_2_	*F*(000) = 1200
*M**_r_* = 295.11	*D*_x_ = 1.556 Mg m^−^^3^
Monoclinic, *P*2_1_/*c*	Mo *K*α radiation, λ = 0.71073 Å
Hall symbol: -P 2ybc	Cell parameters from 5810 reflections
*a* = 17.1738 (14) Å	θ = 2.4–25.3°
*b* = 10.5718 (8) Å	µ = 0.51 mm^−^^1^
*c* = 14.1457 (11) Å	*T* = 150 K
β = 101.192 (5)°	Plate, red
*V* = 2519.4 (3) Å^3^	0.07 × 0.04 × 0.01 mm
*Z* = 8	

### Data collection

**Table:**

Bruker X8 KappaCCD APEXII diffractometer	4600 independent reflections
Radiation source: fine-focus sealed tube	3466 reflections with *I* > 2σ(*I*)
graphite	*R*_int_ = 0.040
ω and φ scans	θ_max_ = 25.4°, θ_min_ = 3.5°
Absorption correction: multi-scan (*SADABS*; Sheldrick, 1997)	*h* = −20→20
*T*_min_ = 0.965, *T*_max_ = 0.995	*k* = −12→11
24324 measured reflections	*l* = −16→17

### Refinement

**Table:**

Refinement on *F*^2^	Primary atom site location: structure-invariant direct methods
Least-squares matrix: full	Secondary atom site location: difference Fourier map
*R*[*F*^2^ > 2σ(*F*^2^)] = 0.032	Hydrogen site location: inferred from neighbouring sites
*wR*(*F*^2^) = 0.085	H atoms treated by a mixture of independent and constrained refinement
*S* = 1.02	*w* = 1/[σ^2^(*F*_o_^2^) + (0.0396*P*)^2^ + 1.1952*P*] where *P* = (*F*_o_^2^ + 2*F*_c_^2^)/3
4600 reflections	(Δ/σ)_max_ = 0.001
355 parameters	Δρ_max_ = 0.24 e Å^−^^3^
6 restraints	Δρ_min_ = −0.24 e Å^−^^3^

### Special details

**Table:**

Geometry. All e.s.d.'s (except the e.s.d. in the dihedral angle between two l.s. planes) are estimated using the full covariance matrix. The cell e.s.d.'s are taken into account individually in the estimation of e.s.d.'s in distances, angles and torsion angles; correlations between e.s.d.'s in cell parameters are only used when they are defined by crystal symmetry. An approximate (isotropic) treatment of cell e.s.d.'s is used for estimating e.s.d.'s involving l.s. planes.
Refinement. Refinement of *F*^2^ against ALL reflections. The weighted *R*-factor *wR* and goodness of fit *S* are based on *F*^2^, conventional *R*-factors *R* are based on *F*, with *F* set to zero for negative *F*^2^. The threshold expression of *F*^2^ > σ(*F*^2^) is used only for calculating *R*-factors(gt) *etc*. and is not relevant to the choice of reflections for refinement. *R*-factors based on *F*^2^ are statistically about twice as large as those based on *F*, and *R*- factors based on ALL data will be even larger.

### Fractional atomic coordinates and isotropic or equivalent isotropic displacement parameters (Å^2^)

**Table:**

	*x*	*y*	*z*	*U*_iso_*/*U*_eq_	
Cl1	0.49916 (4)	0.47039 (5)	0.65986 (4)	0.03327 (15)	
Cl2	0.26299 (4)	0.18196 (7)	0.74680 (4)	0.04150 (17)	
Cl3	−0.05691 (3)	0.62367 (6)	0.53847 (4)	0.03368 (16)	
Cl4	0.12169 (4)	0.98217 (6)	0.40871 (5)	0.04362 (18)	
O1	0.58372 (8)	0.22644 (14)	1.02574 (10)	0.0271 (3)	
O2	0.36779 (9)	0.51109 (15)	1.11813 (10)	0.0308 (4)	
O3	0.13914 (10)	0.96716 (17)	0.83068 (11)	0.0381 (4)	
O4	0.36096 (9)	0.70522 (15)	0.73428 (10)	0.0317 (4)	
N1	0.42445 (10)	0.31116 (17)	0.78706 (12)	0.0254 (4)	
N2	0.49446 (10)	0.36348 (17)	1.12812 (12)	0.0234 (4)	
H2X	0.4811 (12)	0.396 (2)	1.1829 (11)	0.035*	
H2Y	0.5399 (9)	0.3149 (19)	1.1359 (14)	0.035*	
N3	0.06269 (10)	0.83177 (18)	0.56257 (12)	0.0282 (4)	
N4	0.27682 (11)	0.84936 (19)	0.83330 (12)	0.0294 (4)	
H4X	0.3282 (7)	0.826 (2)	0.8623 (15)	0.044*	
H4Y	0.2502 (11)	0.900 (2)	0.8693 (14)	0.044*	
C1	0.37540 (13)	0.3337 (2)	0.69673 (14)	0.0246 (5)	
C2	0.40534 (13)	0.4033 (2)	0.62735 (15)	0.0264 (5)	
C3	0.36328 (15)	0.4175 (2)	0.53380 (15)	0.0324 (6)	
H3	0.3847	0.4657	0.4883	0.039*	
C4	0.28988 (15)	0.3606 (2)	0.50764 (15)	0.0351 (6)	
H4	0.2604	0.3707	0.4439	0.042*	
C5	0.25886 (14)	0.2893 (2)	0.57318 (16)	0.0340 (6)	
H5	0.2086	0.2496	0.5547	0.041*	
C6	0.30200 (13)	0.2760 (2)	0.66665 (15)	0.0293 (5)	
C7	0.41077 (12)	0.36167 (19)	0.86545 (14)	0.0208 (4)	
C8	0.46514 (12)	0.33341 (19)	0.95584 (14)	0.0189 (4)	
C9	0.45032 (12)	0.38251 (19)	1.04179 (14)	0.0205 (4)	
C10	0.37993 (12)	0.4683 (2)	1.04199 (15)	0.0234 (5)	
C11	0.33030 (13)	0.4983 (2)	0.94897 (15)	0.0266 (5)	
H11	0.2869	0.5547	0.9464	0.032*	
C12	0.34440 (12)	0.4484 (2)	0.86732 (15)	0.0241 (5)	
H12	0.3103	0.4699	0.8083	0.029*	
C13	0.53477 (12)	0.25818 (19)	0.95380 (15)	0.0223 (5)	
H13	0.5436	0.2311	0.8927	0.027*	
C14	0.03131 (12)	0.7975 (2)	0.46603 (14)	0.0260 (5)	
C15	−0.02731 (13)	0.7046 (2)	0.44466 (14)	0.0273 (5)	
C16	−0.06283 (14)	0.6751 (2)	0.35062 (15)	0.0312 (5)	
H16	−0.1021	0.6107	0.3381	0.037*	
C17	−0.04074 (14)	0.7399 (2)	0.27537 (15)	0.0340 (6)	
H17	−0.0651	0.7203	0.2109	0.041*	
C18	0.01639 (14)	0.8329 (2)	0.29324 (15)	0.0322 (6)	
H18	0.0317	0.8772	0.2413	0.039*	
C19	0.05143 (13)	0.8613 (2)	0.38746 (16)	0.0295 (5)	
C20	0.13475 (12)	0.8026 (2)	0.60230 (14)	0.0228 (5)	
C21	0.16673 (12)	0.8469 (2)	0.69960 (14)	0.0224 (5)	
C22	0.24313 (12)	0.8152 (2)	0.74440 (14)	0.0228 (5)	
C23	0.29463 (13)	0.7359 (2)	0.69351 (15)	0.0253 (5)	
C24	0.26042 (14)	0.6948 (2)	0.59531 (15)	0.0317 (5)	
H24	0.2919	0.6462	0.5605	0.038*	
C25	0.18613 (13)	0.7242 (2)	0.55375 (15)	0.0286 (5)	
H25	0.1656	0.6936	0.4907	0.034*	
C26	0.11859 (13)	0.9265 (2)	0.74825 (16)	0.0299 (5)	
H26	0.0673	0.9490	0.7140	0.036*	

### Atomic displacement parameters (Å^2^)

**Table:**

	*U*^11^	*U*^22^	*U*^33^	*U*^12^	*U*^13^	*U*^23^
Cl1	0.0426 (3)	0.0302 (3)	0.0295 (3)	0.0019 (3)	0.0133 (3)	−0.0011 (2)
Cl2	0.0421 (4)	0.0542 (4)	0.0276 (3)	−0.0097 (3)	0.0050 (3)	0.0044 (3)
Cl3	0.0358 (3)	0.0457 (4)	0.0196 (3)	−0.0011 (3)	0.0053 (2)	0.0034 (2)
Cl4	0.0342 (3)	0.0497 (4)	0.0439 (4)	−0.0070 (3)	−0.0002 (3)	0.0110 (3)
O1	0.0270 (8)	0.0292 (8)	0.0224 (8)	0.0023 (7)	−0.0018 (7)	0.0026 (7)
O2	0.0319 (9)	0.0377 (9)	0.0247 (8)	0.0008 (7)	0.0101 (7)	−0.0064 (7)
O3	0.0365 (9)	0.0512 (11)	0.0256 (8)	0.0079 (8)	0.0039 (7)	−0.0143 (8)
O4	0.0248 (8)	0.0420 (10)	0.0261 (8)	0.0089 (7)	−0.0003 (7)	0.0023 (7)
N1	0.0298 (10)	0.0297 (10)	0.0165 (9)	0.0040 (8)	0.0036 (8)	0.0028 (8)
N2	0.0255 (10)	0.0270 (10)	0.0174 (9)	−0.0026 (8)	0.0036 (8)	−0.0011 (8)
N3	0.0258 (10)	0.0383 (11)	0.0189 (9)	0.0050 (9)	0.0008 (8)	−0.0034 (8)
N4	0.0281 (10)	0.0381 (12)	0.0190 (9)	0.0055 (9)	−0.0028 (8)	−0.0049 (8)
C1	0.0306 (12)	0.0250 (12)	0.0173 (10)	0.0116 (10)	0.0023 (9)	0.0003 (9)
C2	0.0377 (13)	0.0222 (12)	0.0207 (10)	0.0101 (10)	0.0087 (9)	−0.0006 (9)
C3	0.0524 (16)	0.0274 (13)	0.0182 (11)	0.0140 (12)	0.0093 (10)	0.0022 (10)
C4	0.0501 (16)	0.0367 (14)	0.0155 (10)	0.0157 (12)	−0.0014 (10)	−0.0010 (10)
C5	0.0338 (13)	0.0406 (14)	0.0250 (12)	0.0088 (11)	−0.0007 (10)	−0.0064 (11)
C6	0.0341 (13)	0.0324 (13)	0.0209 (11)	0.0082 (11)	0.0038 (10)	0.0028 (10)
C7	0.0212 (11)	0.0206 (11)	0.0205 (10)	−0.0022 (9)	0.0040 (9)	0.0032 (9)
C8	0.0204 (10)	0.0177 (10)	0.0182 (10)	−0.0027 (8)	0.0027 (8)	0.0023 (8)
C9	0.0209 (10)	0.0197 (11)	0.0206 (10)	−0.0065 (9)	0.0032 (9)	0.0026 (9)
C10	0.0230 (11)	0.0233 (11)	0.0248 (11)	−0.0064 (9)	0.0067 (9)	−0.0017 (9)
C11	0.0230 (11)	0.0273 (12)	0.0288 (11)	0.0018 (10)	0.0033 (9)	0.0004 (10)
C12	0.0246 (11)	0.0236 (11)	0.0224 (11)	0.0023 (9)	0.0002 (9)	0.0019 (9)
C13	0.0261 (11)	0.0194 (11)	0.0212 (10)	−0.0030 (9)	0.0041 (9)	0.0000 (9)
C14	0.0220 (11)	0.0367 (13)	0.0186 (10)	0.0096 (10)	0.0018 (9)	−0.0012 (10)
C15	0.0266 (12)	0.0361 (13)	0.0188 (10)	0.0048 (10)	0.0036 (9)	0.0015 (10)
C16	0.0308 (13)	0.0394 (14)	0.0221 (11)	0.0013 (11)	0.0016 (10)	−0.0003 (10)
C17	0.0362 (13)	0.0468 (15)	0.0164 (10)	0.0046 (12)	−0.0011 (10)	−0.0023 (10)
C18	0.0332 (13)	0.0421 (14)	0.0210 (11)	0.0095 (11)	0.0046 (10)	0.0074 (10)
C19	0.0220 (11)	0.0357 (13)	0.0298 (12)	0.0043 (10)	0.0029 (9)	0.0028 (10)
C20	0.0233 (11)	0.0257 (12)	0.0193 (10)	0.0016 (9)	0.0035 (9)	0.0024 (9)
C21	0.0239 (11)	0.0247 (12)	0.0189 (10)	0.0009 (9)	0.0046 (9)	−0.0011 (9)
C22	0.0229 (11)	0.0256 (11)	0.0194 (10)	−0.0007 (9)	0.0031 (9)	0.0008 (9)
C23	0.0245 (12)	0.0289 (12)	0.0219 (10)	0.0032 (10)	0.0029 (9)	0.0029 (9)
C24	0.0323 (13)	0.0386 (14)	0.0234 (11)	0.0126 (11)	0.0032 (10)	−0.0069 (10)
C25	0.0321 (12)	0.0335 (13)	0.0188 (10)	0.0060 (10)	0.0015 (9)	−0.0054 (10)
C26	0.0245 (12)	0.0380 (14)	0.0265 (12)	0.0036 (10)	0.0030 (9)	−0.0023 (11)

### Geometric parameters (Å, °)

**Table:**

Cl1—C2	1.738 (2)	C7—C12	1.467 (3)
Cl2—C6	1.737 (2)	C8—C9	1.390 (3)
Cl3—C15	1.736 (2)	C8—C13	1.441 (3)
Cl4—C19	1.743 (2)	C9—C10	1.512 (3)
O1—C13	1.234 (2)	C10—C11	1.457 (3)
O2—C10	1.223 (2)	C11—C12	1.334 (3)
O3—C26	1.229 (3)	C11—H11	0.9500
O4—C23	1.217 (2)	C12—H12	0.9500
N1—C7	1.293 (3)	C13—H13	0.9500
N1—C1	1.408 (3)	C14—C15	1.397 (3)
N2—C9	1.322 (2)	C14—C19	1.400 (3)
N2—H2X	0.917 (9)	C15—C16	1.387 (3)
N2—H2Y	0.922 (9)	C16—C17	1.380 (3)
N3—C20	1.293 (3)	C16—H16	0.9500
N3—C14	1.414 (3)	C17—C18	1.377 (3)
N4—C22	1.328 (3)	C17—H17	0.9500
N4—H4X	0.931 (9)	C18—C19	1.385 (3)
N4—H4Y	0.921 (9)	C18—H18	0.9500
C1—C6	1.390 (3)	C20—C21	1.456 (3)
C1—C2	1.402 (3)	C20—C25	1.474 (3)
C2—C3	1.387 (3)	C21—C22	1.383 (3)
C3—C4	1.381 (3)	C21—C26	1.444 (3)
C3—H3	0.9500	C22—C23	1.501 (3)
C4—C5	1.380 (3)	C23—C24	1.465 (3)
C4—H4	0.9500	C24—C25	1.334 (3)
C5—C6	1.392 (3)	C24—H24	0.9500
C5—H5	0.9500	C25—H25	0.9500
C7—C8	1.460 (3)	C26—H26	0.9500
			
C7—N1—C1	122.17 (18)	C7—C12—H12	118.8
C9—N2—H2X	122.1 (13)	O1—C13—C8	124.65 (19)
C9—N2—H2Y	121.0 (13)	O1—C13—H13	117.7
H2X—N2—H2Y	116.9 (14)	C8—C13—H13	117.7
C20—N3—C14	120.77 (18)	C15—C14—C19	116.48 (19)
C22—N4—H4X	123.3 (13)	C15—C14—N3	120.83 (19)
C22—N4—H4Y	120.7 (13)	C19—C14—N3	122.5 (2)
H4X—N4—H4Y	116.0 (14)	C16—C15—C14	122.0 (2)
C6—C1—C2	116.73 (19)	C16—C15—Cl3	118.86 (18)
C6—C1—N1	123.4 (2)	C14—C15—Cl3	119.13 (16)
C2—C1—N1	119.2 (2)	C17—C16—C15	119.5 (2)
C3—C2—C1	122.0 (2)	C17—C16—H16	120.2
C3—C2—Cl1	119.52 (18)	C15—C16—H16	120.2
C1—C2—Cl1	118.43 (16)	C18—C17—C16	120.4 (2)
C4—C3—C2	119.2 (2)	C18—C17—H17	119.8
C4—C3—H3	120.4	C16—C17—H17	119.8
C2—C3—H3	120.4	C17—C18—C19	119.5 (2)
C5—C4—C3	120.6 (2)	C17—C18—H18	120.2
C5—C4—H4	119.7	C19—C18—H18	120.2
C3—C4—H4	119.7	C18—C19—C14	122.1 (2)
C4—C5—C6	119.3 (2)	C18—C19—Cl4	118.83 (18)
C4—C5—H5	120.3	C14—C19—Cl4	119.07 (17)
C6—C5—H5	120.3	N3—C20—C21	119.18 (19)
C1—C6—C5	122.1 (2)	N3—C20—C25	122.72 (19)
C1—C6—Cl2	119.52 (16)	C21—C20—C25	118.09 (18)
C5—C6—Cl2	118.42 (19)	C22—C21—C26	120.17 (19)
N1—C7—C8	118.35 (18)	C22—C21—C20	120.36 (19)
N1—C7—C12	123.02 (19)	C26—C21—C20	119.46 (18)
C8—C7—C12	118.62 (18)	N4—C22—C21	124.6 (2)
C9—C8—C13	121.14 (18)	N4—C22—C23	114.66 (18)
C9—C8—C7	119.67 (18)	C21—C22—C23	120.70 (18)
C13—C8—C7	119.16 (17)	O4—C23—C24	122.5 (2)
N2—C9—C8	125.56 (19)	O4—C23—C22	120.41 (19)
N2—C9—C10	113.89 (18)	C24—C23—C22	117.07 (18)
C8—C9—C10	120.53 (18)	C25—C24—C23	121.5 (2)
O2—C10—C11	123.0 (2)	C25—C24—H24	119.3
O2—C10—C9	119.78 (19)	C23—C24—H24	119.3
C11—C10—C9	117.17 (18)	C24—C25—C20	122.3 (2)
C12—C11—C10	121.5 (2)	C24—C25—H25	118.9
C12—C11—H11	119.2	C20—C25—H25	118.9
C10—C11—H11	119.2	O3—C26—C21	124.8 (2)
C11—C12—C7	122.34 (19)	O3—C26—H26	117.6
C11—C12—H12	118.8	C21—C26—H26	117.6
			
C7—N1—C1—C6	−77.3 (3)	C20—N3—C14—C15	110.1 (2)
C7—N1—C1—C2	111.9 (2)	C20—N3—C14—C19	−75.8 (3)
C6—C1—C2—C3	1.7 (3)	C19—C14—C15—C16	1.2 (3)
N1—C1—C2—C3	173.0 (2)	N3—C14—C15—C16	175.7 (2)
C6—C1—C2—Cl1	−176.76 (16)	C19—C14—C15—Cl3	−178.67 (16)
N1—C1—C2—Cl1	−5.4 (3)	N3—C14—C15—Cl3	−4.2 (3)
C1—C2—C3—C4	−0.4 (3)	C14—C15—C16—C17	−0.8 (3)
Cl1—C2—C3—C4	177.98 (17)	Cl3—C15—C16—C17	179.07 (18)
C2—C3—C4—C5	−0.8 (3)	C15—C16—C17—C18	0.3 (4)
C3—C4—C5—C6	0.7 (3)	C16—C17—C18—C19	−0.2 (3)
C2—C1—C6—C5	−1.8 (3)	C17—C18—C19—C14	0.7 (3)
N1—C1—C6—C5	−172.7 (2)	C17—C18—C19—Cl4	−178.31 (18)
C2—C1—C6—Cl2	177.02 (16)	C15—C14—C19—C18	−1.1 (3)
N1—C1—C6—Cl2	6.1 (3)	N3—C14—C19—C18	−175.5 (2)
C4—C5—C6—C1	0.7 (3)	C15—C14—C19—Cl4	177.85 (17)
C4—C5—C6—Cl2	−178.15 (17)	N3—C14—C19—Cl4	3.5 (3)
C1—N1—C7—C8	−179.69 (19)	C14—N3—C20—C21	175.9 (2)
C1—N1—C7—C12	−1.3 (3)	C14—N3—C20—C25	−5.1 (3)
N1—C7—C8—C9	−177.60 (19)	N3—C20—C21—C22	178.9 (2)
C12—C7—C8—C9	3.9 (3)	C25—C20—C21—C22	−0.2 (3)
N1—C7—C8—C13	4.4 (3)	N3—C20—C21—C26	−2.5 (3)
C12—C7—C8—C13	−174.09 (18)	C25—C20—C21—C26	178.5 (2)
C13—C8—C9—N2	−2.5 (3)	C26—C21—C22—N4	2.5 (3)
C7—C8—C9—N2	179.58 (19)	C20—C21—C22—N4	−178.8 (2)
C13—C8—C9—C10	175.95 (18)	C26—C21—C22—C23	−178.1 (2)
C7—C8—C9—C10	−2.0 (3)	C20—C21—C22—C23	0.5 (3)
N2—C9—C10—O2	−1.2 (3)	N4—C22—C23—O4	1.6 (3)
C8—C9—C10—O2	−179.80 (19)	C21—C22—C23—O4	−177.8 (2)
N2—C9—C10—C11	177.42 (18)	N4—C22—C23—C24	179.7 (2)
C8—C9—C10—C11	−1.2 (3)	C21—C22—C23—C24	0.3 (3)
O2—C10—C11—C12	−178.9 (2)	O4—C23—C24—C25	176.6 (2)
C9—C10—C11—C12	2.5 (3)	C22—C23—C24—C25	−1.5 (3)
C10—C11—C12—C7	−0.6 (3)	C23—C24—C25—C20	1.9 (4)
N1—C7—C12—C11	178.9 (2)	N3—C20—C25—C24	179.9 (2)
C8—C7—C12—C11	−2.7 (3)	C21—C20—C25—C24	−1.0 (3)
C9—C8—C13—O1	3.6 (3)	C22—C21—C26—O3	−2.6 (4)
C7—C8—C13—O1	−178.44 (19)	C20—C21—C26—O3	178.7 (2)

### Hydrogen-bond geometry (Å, °)

**Table:**

*D*—H···*A*	*D*—H	H···*A*	*D*···*A*	*D*—H···*A*
N2—H2X···Cl1^i^	0.92 (1)	2.60 (1)	3.4590 (18)	156.(2)
N2—H2Y···O1	0.92 (1)	2.08 (2)	2.722 (2)	126.(2)
N2—H2Y···O4^i^	0.92 (1)	2.26 (2)	2.933 (2)	130.(2)
N4—H4X···O1^i^	0.93 (1)	2.04 (1)	2.916 (2)	155 (2)
N4—H4Y···O3	0.92 (1)	2.01 (2)	2.666 (3)	127.(2)

Symmetry codes: (i) −*x*+1, −*y*+1, −*z*+2.
